# Idiopathic Pulmonary Fibrosis Serum proteomic analysis before and after nintedanib therapy

**DOI:** 10.1038/s41598-020-66296-z

**Published:** 2020-06-10

**Authors:** Claudia Landi, Laura Bergantini, Paolo Cameli, Miriana d’Alessandro, Alfonso Carleo, Enxhi Shaba, Paola Rottoli, Luca Bini, Elena Bargagli

**Affiliations:** 1 Functional Proteomics Lab, Dept. Life Sciences, University of Siena, Siena, Italy; 2 Respiratory Diseases and Lung Transplant Unit, Dept. Internal and Specialist Medicine, AOUS, Siena, Italy; 3 Department of Pneumology, Medical School Hannover (MHH), Hannover, Germany

**Keywords:** Biochemistry, Computational biology and bioinformatics, Systems biology

## Abstract

Idiopathic pulmonary fibrosis (IPF) is a fatal progressive disease with a median survival of 2–5 years. Nintedanib is a small tyrosine kinase inhibitor that reduces IPF progression, significantly slowing the annual decline in Forced Vital Capacity (FVC). Very little data is available on the molecular mechanisms of this treatment in IPF, despite a growing interest in the definition of IPF pathogenesis and target therapy. A functional proteomic approach was applied to the analysis of serum samples from IPF patients in order to highlight differential proteins potentially indicative of drug-induced molecular pathways modifications and response to therapy. Twelve serum samples were collected from six IPF patients in care at Siena Regional Referral Center for Interstitial Lung Diseases (ILDs) and treated with nintedanib for one year. Serum samples were analyzed at baseline (T0 before starting therapy) and after one year of treatment (T1) and underwent differential proteomic and bioinformatic analysis. Proteomic analysis revealed 13 protein species that were significantly increased after one year of treatment. When the targets of nintedanib (VEGFR, FGFR and PDGFR) were added, enrichment analysis extracted molecular pathways and process networks involved in cell differentiation (haptoglobin and albumin), coagulation (antithrombin III), epithelial mesenchymal transition, cell proliferation and transmigration. PI3K and MAPK induced up-regulation of apolipoprotein C3. Proteomic study found 13 protein species up-regulated in IPF patients after one year of nintedanib treatment. Haptoglobin, a central hub of our analysis was validated by 2D-WB and ELISA as theranostic marker in a more numerous populations of patients.

## Introduction

Idiopathic pulmonary fibrosis (IPF) is a disabling fatal progressive disease of unknown etiology characterized by HRCT evidence *usual interstitial pneumonia* (UIP) pattern in the lungs^1,2^. In this disease, fibroblast and myofibroblast deposition in the alveolar walls is associated with overproduction of extracellular matrix. IPF pathogenesis is not completely understood, rare and common genetic variants (surfactant A and C, MUC5B, TERT or TERC polymorphism) have been associated with sporadic and familiar forms of pulmonary fibrosis. Moreover, occupational and environmental risk factors (metal dust, pollution, farming, etc…) for IPF have been evaluated^1,2^.

The standard goal of IPF treatment is currently to stabilize or reduce the rate of disease progression, since no specific treatment is known to modify its natural progression. Two antifibrotic therapies, nintedanib and pirfenidone, were recently approved and both drugs have been a breakthrough in the management of IPF^3^. nintedanib is a tyrosine kinase inhibitor that reduces IPF progression, significantly slowing the annual decline in FVC. Already applied in oncology, very little data is available on the molecular mechanisms of this treatment in IPF, despite a growing interest in the definition of specific prognostic biomarkers of drug response. Recent studies identified a certain number of kinases as target of nintedanib in *in vitro* mechanism of action^4,5^.

The development of therapeutic agents for IPF has created an urgent need for biomarkers to monitor disease^6^. In fact, the difficulty of early diagnosis and of differentiating IPF from various idiopathic interstitial pneumonias, as well as the impossibility of predicting patient outcome, have prompted research into biomarkers. Useful biomarkers have to be specific, sensitive and readily detectable in biological fluids by reproducible non-invasive procedures. Current prognostic biomarkers of treatment response in IPF are based on clinical and functional parameters. In particular, GAP score, FVC and DLCO percentages predict disease progression and mortality but no biochemical indicator that can recognize response to antifibrotic treatment has yet been identified. The pace of biomarker discovery accelerated with the advent of the -omics sciences (including proteomics) that produce a vast amount of data and hold promise for personalized care.

In this context, we applied a functional proteomic approach to the analysis of serum samples from IPF patients before and after one year of nintedanib therapy in order to highlight biomarkers indicative of drug-induced molecular pathway modifications and response to therapy. Starting from already known nintedanib target molecules (VEGFR, PDGFR and FGFR), we identified metabolic pathways that could be targeted by nintedanib and that may be mirrored at serum. Through this study, we hoped to obtain a clearer picture by combining proteomic, clinical and molecular data.

## Materials and Methods

### Population

Six IPF patients (4 males, mean age 79 ± 5.6 years, 3 ex-smokers and 1 no-smoker; 2 females, mean age 69 ± 4, 1 ex-smoker) were enrolled in the study. All the patients had a definitive diagnosis of IPF based on multidisciplinary discussion (MDD) with no features for other ILDs. The patients were diagnosed according to ATS/ERS guidelines at Siena Regional Referral Centre for Interstitial Lung Diseases. They were selected among treatment naïve patients at the moment of the diagnosis, underwent nintedanib treatment according to the italian national drug inclusion criteria. None of them were treated before with pirfenidone or other anti-fibrotic drugs. Demographic data, smoking habits, onset symptoms and comorbidities were recorded in a database together with BAL differential cell count, lung function tests and blood gas analysis results. Biomarker research was performed by serum analysis of KL-6. Functional, radiological, histological and immunological data was also collected. Lung function tests were performed according to ATS/ERS guidelines to obtain FEV1, FVC, TLC and DLCO percentages^7^. High-resolution computed tomography of the chest (HRCT) was performed in all patients and interpreted by experienced radiologists. Diagnosis was formulated in a context of multidisciplinary discussion. All patients gave written informed consent to participation in the study, which was approved by the local ethics committee C.E.A.V.S.E. (code number 180712). After informed consent of the patients, bronchoalveolar lavage was performed for diagnostic purposes for excluding other interstitial lung diseases. Patients with IPF were treated with the antifibrotic drug nintedanib according to Italian national drug inclusion/exclusion criteria. Nintedanib inclusion criteria in Italy are: age ≥40 years, diagnosis of idiopathic pulmonary fibrosis according to international guidelines, FVC > 50% of predicted and DLCO > 30%. Exclusion criteria for nintedanib treatment in Italy are: ALT, AST > 1.5 × ULN, total bilirubin> 1.5 × ULN, high risk of bleeding, INR > 2, PT, PTT > 150% of ULN, major surgery scheduled in the next 3 months or high risk of thrombosis. Serum from selected patients was drawn at time 0 before starting nintedanib and after 1 year of therapy. Patients were monitored every 3 months. We exclude current smokers and histories of concomitant pathologies (such as cancer, pulmonary hypertension and metabolic disorders). All patients tolerated nintedanib therapy at a stable full dose of 150 mg twice a day for one year. All data was expressed as mean ± standard deviation. Spearman correlation was used for KL-6 analysis. Statistical significance was set at p < 0.05 (GraphPad Prism software 6.0).

### Functional proteomic analysis

Two dimensional analysis of serum samples (Supplementary Material S1) were assessed as reported by Bianchi *et al*.^8^. Spots were considered differentially abundant when the ratio of mean percentage of relative volumes (%V) was greater than 1.5. The Mann Whitney test (p ≤ 0.05) was used to determine whether the data sets were significantly different from each other (Graphpad Prism 6.0). Differential spots were used to perform multivariate Principal Component Analysis (PCA), simplifying the amount of data (%V variables) by linear transformation. PCA plotted experimental groups in a two-dimensional plane on the basis of differential spot patterns. Differential spots were also used to perform cluster analysis by Cluster 3.0 (Java tree view). Differential spots were identified by Peptide Mass Fingerprinting (PMF) with a MALDI-ToF mass spectrometer (Bruker Corporation, Billerica, MA, United States). The proteins identified were used to perform functional and pathway analysis with Metacore software version 6.8. Haptoglobin abundance was validated by 2D western blot and ELISA analysis in a new cohort of samples. For more details, see Supplementary Material S2.

### Significance

- Serum Proteomic analysis highlights biomarkers indicative of drug-induced molecular pathway modifications in response to therapy.
- Modified metabolic pathways targeted by nintedanib are mirrored at serum level.
- Serum haptoglobin as biomarkers was further analyzed by 2D-WB and ELISA test in a wider number of samples.

## Results

### Population

Details of our population are reported in Table 1. The age of patients was quite high because nintedanib is approved also for older patients in Italy. The patients showed restrictive deficits in the lung function tests, as expected. They showed a mild increase in neutrophils and eosinophils in BAL and were not positive for autoantibodies. The KL-6 levels were reported in Table 1. Positive correlation was found among KL-6 levels a baseline and BAL lymphocytes percentages (r = 0.74; p = 0.03).

**Table 1:** Demographic and clinical data of the six IPF patients in our study.

		Average	SD	
**A**				
Macrophages		49,2	20,8	
Lymphocytes		21,8	16,5	
Neutrophis		23,6	9,3	
Eosinophils		5,4	4,9	
Age		79,0	5,7	
Gender		4 Male/2 Female		
Smoking history		2 Never/4 Former		
	**T0**	**T1**	**FC(T1/T0)**	**p value**
**B**				
FEV1	78.3 ± 14.6	78.8 ± 19.6	1,01	ns
FVC	75.3 ± 18.6	76.0 ± 19.6	1,01	ns
DLCO (%)	39.0 ± 25.2	36.7 ± 16.6	0,94	ns
PaO_2_%	67.7 ± 6.9	69.4 ± 12.4	1,03	ns
PaCO_2_%	38.2 ± 1.9	40.1 ± 3.6	1,05	ns
KL-6 (U/ml)	1721.7 ± 510.5	1360.3 ± 680	0,79	ns

Table 1A reported the differential BAL cell count in percentage. Table 1B showed Pulmonary function test, emogas analysis, and serum levels of KL-6 protein before (T0) and after 1 year of nintedanib treatment (T1). The average (±standard deviation) value are reported per each groups, as well as the FoldChange (FC) and the Wilxocon test significativity (p value).

### Proteomic analysis of sera from IPF patients before and after 12 months of nintedanib treatment

**Figure 1:**
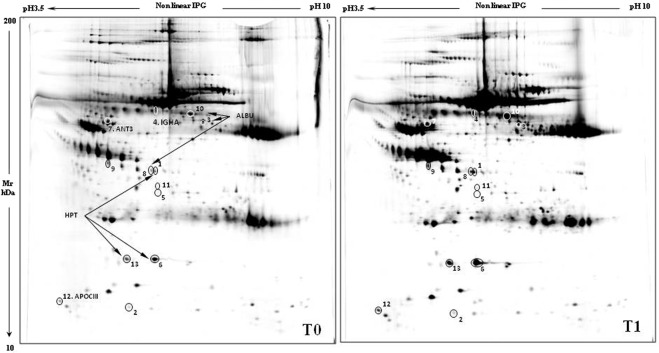
Gel images reporting the Master gel of T0 and T1 conditions. Numbers and circles highlight the differential spots found corresponding to that in Table 2. Master gel T0 also reports the name of the identified proteins.

**Figure 2:**
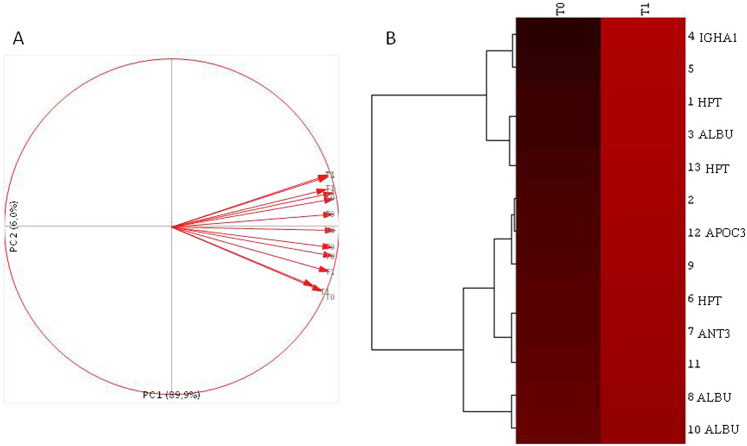
(**A**) PCA graph obtained with the %V of the differential spots found between T0 and T1 samples. (**B**) Cluster analysis of the normalized values of the %V means of the differential spots found in T0 vs T1 samples.

Proteomic analysis of sera was performed by 2DE, extracting characteristic protein patterns from T0 (before treatment) and T1 (after 12 months of treatment) samples. The gel images showed an average of 1170 spots. They were compared by image analysis, obtaining 13 abundant distinctive differential spots by the Mann Whitney test (Fig. 1). Eight of these spots were identified by mass spectrometry (MALDI-ToF–ToF/MS) and were found to match five proteins: albumin (ALBU), haptoglobin (HPT), antithrombin III (ANT3), immunoglobulin heavy constant alpha 1 (IGHA1) and apolipoprotein CIII (APOC3). The protein identification and statistical results are reported in Table 2 (spot numbers are those in Fig. 1). The %V of the differential spots in each gel were used to build a matrix with the 13 spots in the rows and the 12 gels in the columns. Interpretation of this matrix by Principal Component Analysis highlighted the spatial distributions of the 12 samples (6 T0 and 6 T1) in the plot of Fig. 2A. The first and second principal components (PC1 and PC2) explained 89.9% and 6% of the variance, respectively, showing a homogeneous distribution of the differential spots between samples before and after the treatment. Indeed, all samples fell in the right part of the graph, without any distinct grouping for T0 and T1. Cluster analysis gave us an instant visualization of the spot abundance trend before and after treatment. Figure 2B shows that nintedanib induced up-regulation of all protein spots.

**Table 2:** Protein identification results.

Spot Number^a^	Protein Name	Abbreviation^b^	Accession^c^	Mean T0	Mean T1	T0/T1	T1/T0	Wilcoxon	FDR	Mascot Search Results^d^		
										Score	Coverage (%)	Matched Peptides
1	Haptoglobin	HPT	P00738	0,033 ± 0,02	0,095 ± 0,027	0.346	2.89	2.16E-03	6.49E-03	218	33	17
3	Albumin	ALBU	P02768	0,022 ± 0,019	0,059 ± 0,018	0.366	2.73	2.16E-03	6.49E-03	114	18	11
4	Immunoglobulin heavy constant alpha 1	IGHA1	P01876	0,042 ± 0,031	0,186 ± 0,104	0.227	4.4	8.66E-03	2.60E-02	155	29	12
6	Haptoglobin	HPT	P00738	0,281 ± 0,06	0,528 ± 0,183	0.532	1.88	8.66E-03	2.60E-02	143	27	11
7	Antithrombin-III	ANT3	P01008	0,078 ± 0,028	0,136 ± 0,036	0.571	1.75	8.66E-03	2.60E-02	107	23	10
8	Albumin	ALBU	P02768	0,069 ± 0,017	0,107 ± 0,029	0.64	1.56	1.52E-02	4.55E-02	76	17	9
12	Apolipoprotein C-III	APOC3	P02656	0,027 ± 0,011	0,053 ± 0,025	0.505	1.98	1.52E-02	4.55E-02	64	23	3
13	Haptoglobin	HPT	P00738	0,079 ± 0,027	0,193 ± 0,117	0.411	2.44	1.52E-02	4.55E-02	100	18	8

^a^Spot number match those reported in the representative two dimensional (2D)- gels shown in Fig. 1.

^b^Identifier (ID) referred to UniProt.

^c^Accession number referred to UniProt.

^d^MASCOT search results with number of matched peptides, sequence coverage % (number of the identified residues/total number of amino acid residues in the protein sequence) (Matrix Science, London, UK; http://www.matrixscience.com). Moreover, statistical analysis results are reported including Means and deviations standard, %V mean ratios and significant Mann-Whitney non-parametrical test p value (p < 0.05) between the analyzed conditions.

Table 2 also reports the statistical analysis results such as Wilcoxon test and FDR.

#### Protein network results

**Figure 3:**
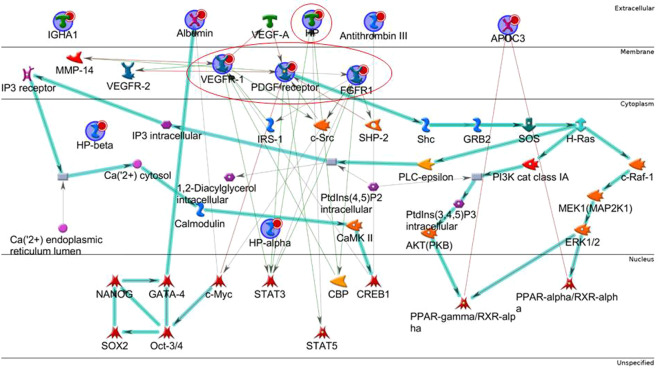
Protein network by MetaCore built with the differential proteins found (blue spheres) adding FGFR1, VEGFR-1, PDGF receptor, known to be targets of the Nintedanib treatment. PDGF-R-beta, FGFR1, VEGFR-1, HP, PDGF receptor result central hubs. Green edges indicate «induction», red edges are correlated with inhibition, while grey edges indicate an unspecified effect. Bold light blue line represent well-known canonical pathways.

The protein network of the five differential proteins built by MetaCore software using the shortest path algorithm immediately suggested an absence of direct interaction between them (data not shown). Considering the central role of FGFR1, VEGFR and PDGFR in the mechanism of action of nintedanib, we decided to add these proteins to the list of differential proteins evaluated by MetaCore. An interesting protein network emerged (Fig. 3): PDGF-R-beta, FGFR1, VEGFR-1, HPT and PDGF were central hubs, i.e. proteins with many interactions and a potentially central role in the network. The pale blue line in Fig. 3 highlights the canonical pathways obtained with the uploaded proteins: starting from PDGFR, following the arrows, the signal transduction cascade modulates several transcription factors, such as PPAR-γ/RXR-alpha and PPAR-α/RXR-alpha, which in turn modulate APOC3, which proved to be abundant in serum of IPF patients after one year of nintedanib treatment. The pathway also leads to CREB1, a key protein regulating ANT3 and VEGFR-1 expression. Another canonical pathway in our protein network, which starts from c-Myc and leads through Oct3/4, SOX2, NANOG and GATA-4, controls albumin expression. Interestingly, another transcriptional factor, STAT3, appears to be involved in the protein network. STAT3 is induced by the principal targets of nintedanib, i.e. FGFR1, VEGFR-1 and PDGFR, and also by HPT.

Since the importance of haptoglobin, considered as central hub of the network analysis, we decided to perform a validation analysis of this protein by an ELISA assay and by a two-dimensional western blot.

**Figure 4:**
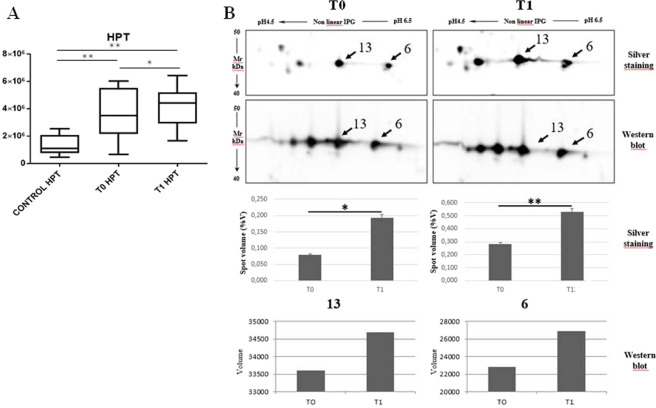
(**A**) Histogram reporting the results obtained by ELISA analysis on 14 patients to T0 and T1 and 14 healthy subjects. **p < 0.01, *p < 0.05 (**B**) Cropped images of Two-dimensional western blot analysis. Images focalize the attentions on the spots corresponding to that differentially abundant in 2D-gels. 2D WB data of haptoglobin (3 minutes of exposition for both the conditions T0 and T1) confirm the obtained proteomic data for the spots 6 and 13. *p < 0.05, **p < 0.01. Original images of 2D WB are in supplementary materials.

The ELISA assay, performed in a new cohort composed of 10 healthy samples and 14 IPF, before and after 1 year of treatment, confirmed the high abundance of haptoglobin in the serum of the patients after one year of treatment with nintedanib (healthy 1,3 × 10^6^ ± 0,2 × 10^6^; T0 3,6 × 10^6^ ± 1,7 × 10^6^; T1 4,2 × 10^6^ ± 1,4 × 10^6^ ng/ml). In particular, the analysis performed by two dimensional WB confirm the up-regulation of the spots 6 and 13 obtained by differential proteomic analysis, as showed in Fig. 4. HPT concentration at the baseline correlated with the percentages of FEV1 (R = −0.67; p = 0.03) and FVC (R = −0.67; p = 0.04).

### Enrichment analysis: pathway maps and process networks

**Figure 5:**
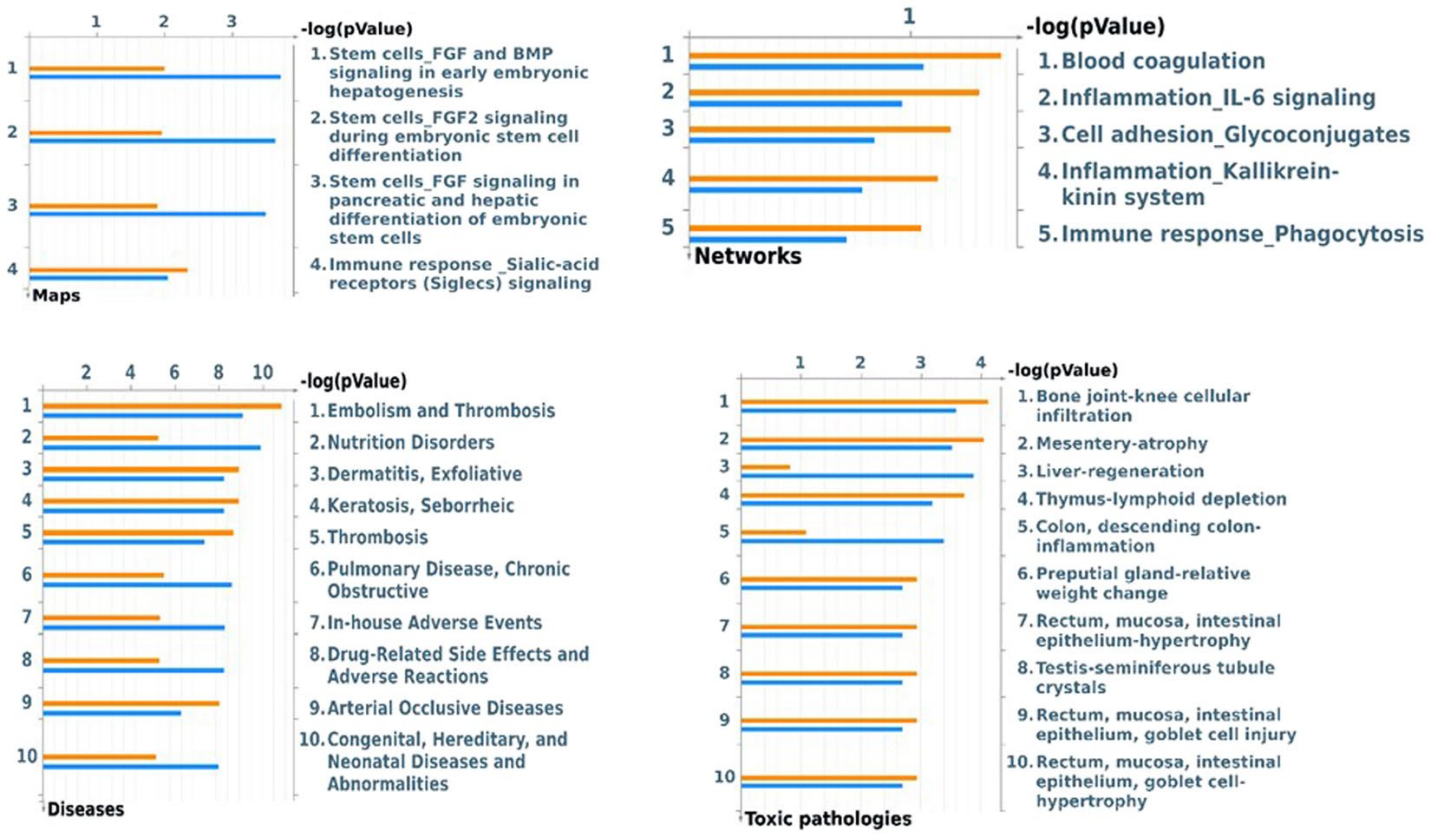
Pathway Maps, Process networks, Disease (by biomarkers) and Toxic pathologies analyses comparison by MetaCore software. Orange histograms represent the p value of the analyses performed with the differential proteins found between T0 and T1 samples (group A). The light blue bars represent the p value of the analyses obtained uploading together the differential proteins found and the targets of Nintedanib: PDGFR, FGFR1, VEGFR (group B).

In order to determine whether the differential proteins found are implicated in mechanisms involving the targets of nintedanib, we compared two enrichment analysis groups (Fig. 5): Group A consisting only of the differential proteins found (orange bars), and Group B consisting of the differential proteins plus the three principal targets of nintedanib (FGFR1, VEGFR-1 and PDGFR; pale blue bars). Figure 5 shows the pathway maps and process network results.

The first three pathway maps obtained were “FGF and BMP signaling in early embryonic hepatogenesis”, “FGF2 signaling during embryonic stem cell differentiation” and “FGF signaling in pancreatic and hepatic differentiation of embryonic stem cells”. These pathways were more significantly associated with Group B (pale blue bars) than Group A. The graphic schemes of these molecular pathways, obtained with MetaCore software, are reported in Supplementary Fig. 1A–C. Other pathway maps had similar importance in the two groups and included: “immune response by sialic acid receptor signaling (Siglecs)” (Supplemental Fig. 1D), “role of ZNF202 in the regulation of atherosclerosis-related genes”, “fenofibrate in treatment of type 2 diabetes and metabolic syndrome X”, “RXR-dependent regulation of lipid metabolism via PPAR, RAR and VDR”, “development by transcription factors in segregation of hepatocytic lineage”, “role of IL-6 in obesity and type 2 diabetes adipocytes”, “self-renewal and pluripotency maintenance of human embryonic stem cells” and “IL-6 induced acute-phase response in hepatocytes and blood coagulation”. Protein network results significantly associated with group A (orange bars) were “hypercoagulation”, “IL-6 signaling in inflammation”, “cell adhesion by glycoconjugates” and “kallikrein kinin system in inflammation and phagocytosis”.

### Biomarkers and toxic pathologies

The results obtained from disease biomarker and toxic pathology evaluations were included in this enrichment analysis. In the first analysis, we obtained pathologies where the proteins uploaded by MetaCore software proved to be potential biomarkers. In the second analysis, we obtained possible toxic pathologies induced by the uploaded proteins. These results are summarized in Fig. 5. Among the diseases recognized there were pulmonary embolism and vascular thrombosis, nutrition disorders, exfoliative dermatitis, seborrheic keratosis, chronic obstructive pulmonary disease, drug-related side effects and adverse reactions. Comparison of the two groups of proteins showed similar results, although nutrition disorders, pulmonary diseases and drug related side effects were prevalent in Group B (pale blue bars).

The toxic pathologies suggested by the uploaded proteins were “bone joint-knee cellular infiltration”, “mesentery atrophy”, “thymus-lymphoid depletion”, “descending colon inflammation” and “cell hypertrophies of intestinal epithelium”, “nose olfactory epithelium and adrenal cortex”, “toxic pathologies of seminiferous tubules”, “glandular stomach-parietal cell depletion” and “splenic lymphoid depletion” (Fig. 5). The proteins of Group B (light blue bars) were associated with “liver regeneration” and “descending colon inflammation” (7–25).

## Discussion

Nintedanib is a pharmacological treatment with anti-fibrotic, anti-proliferative and anti-inflammatory properties used to treat lung cancer and IPF^9^. It was observed that patients with mild-to-moderate IPF, under therapy, have a reduced rate of decline of forced vital capacity (FVC) and disease progression. Adverse events such as acute exacerbations, are also reduced by this drug^10^. Recent literature reports the therapeutic implications of nintedanib, alone or in association with other drugs, for lung cancer and IPF^11,12^, although few cells and molecular targets of the drug have been explored in depth in either diseases. In the present study, we used a top-down proteomic approach, combined with bioinformatics, to study the effects of nintedanib treatment at serum level in IPF patients before and after one year of treatment. Our results highlight possible biomarkers indicative of drug-induced molecular pathway modifications, mirrored at serum level.

Differential proteomic analysis shed light on 13 spots, all increased in abundance after one year of treatment: they were proteoforms of albumin, haptoglobin, antithrombin III, apolipoprotein CIII and immunoglobulin heavy constant alpha 1. However, PCA demonstrated the homogeneity and limited variance of our data, showing that the samples obtained before and after treatment had similar quantitative protein patterns.

Since nintedanib is a multi-tyrosine kinase inhibitor and is known to inhibit PDGFR, VEGFR and FGFR, we merged these proteins with the differential proteins found and we performed MetaCore enrichment analysis. We were interested in discovering any direct or indirect correlations between the targets of nintedanib and the proteins that proved to be modified in serum. Protein network analysis immediately highlighted possible causes of the abundant presence of these five proteins in serum, showing that all proteins were related, save IGHA1. As expected, PDGFR, VEGFR and FGFR emerged as central hubs together with HPT. In inflammatory conditions, HPT is normally up-regulated as acute-phase protein that counteracts inflammation^13^. From the protein network, we can observe HPT, potentially modulated by the action of nintedanib, which acting on its targets, induces STAT3 modulation, as also reported by Uskoković *et al*.^14^. In turn, HPT and STAT3 interaction signaling is involved in the development of fibrosis and in the regulation of fibroblast senescence^14–16^. Interestingly, it has been demonstrated that the inhibition and activation of STAT3 are both related to the fibrosis onset following the cross-talk between STAT3 and TGF-β signaling, that is organ and tissue-dependent^17^. HPT could also be induced by the interaction of FGFR and VEGFR with CREB-binding protein (CBP), which is involved in cell growth, transformation and development and also shows histone acetyltransferase activity^18^. CBP acts on β-catenin in different ways: (i) acetylating β-catenin at lysine 49 in order to negatively regulate the transcription of genes such as c-myc, considered an oncogene and (ii) acting positively to co-activate β-catenin-dependent transcription^18^. This mechanism is closely related to the WNT/β-catenin signaling pathway which is known to play an important role in the pathogenesis of idiopathic pulmonary fibrosis and lung cancer^19–21^. Both these diseases share common pathways and can be treated with nintedanib^11,12^. As suggested by our pathway map analysis, up-regulation of HPT could also be related to “immune response by sialic acid receptor signaling (Siglecs)”. Since the central role of HPT after nintedanib treatment, we decided to validate by ELISA test the behavior of this protein in a wider cohort of samples. Our results confirm the proteomic data, showing an up-regulation of HPT in serum after 1 year of treatment. In particular, 2D-WB highlighted the up-regulation of the two protein species 6 and 13. The graphic scheme in the supplementary material shows that “Siglecs” path may not only be activated by up-regulation of HPT after nintedanib treatment, but also by mucin 1, renamed KL-6 (Fig. S-1D)^22^. This observation prompted us to quantify KL-6 trends after nintedanib treatment^23^. We found a decreasing trend of protein abundance in serum. The correlation, observed in our study, between KL-6 and BAL lymphocytosis, is in line with this finding. KL-6 is normally present in serum due to the permeability of the air–blood barrier and our results suggest that nintedanib treatment could attenuates its production or its transition. Serum KL-6 is normally related with the BALF/serum albumin ratio, a marker of the alveolar–capillary permeability^24^.

Interestingly, differential proteomic analysis revealed an abundance of some protein species of albumin in serum (spots 3 and 8). Enrichment analysis suggested that albumin could be modulated by c-Myc activity through Oct3/4, SOX2, NANOG and GATA-4. PDGFR signaling, a target of nintedanib, is a key regulator of Oct4 and NANOG interfering with mesenchymal stem cell potency^25^. Erdélyi-Belle *et al*. reported that the progressive down-regulation of the *Oct3/4* and *Nanog* genes and progressive up-regulation of albumin lead the human embryonic stem cells to differentiate into the hepatic lineage. For this reason, albumin is also considered a liver-associated gene^26^. Pathway map analysis indicated that albumin is fundamental for the three molecular pathways reported as “FGF and BMP signaling in early embryonic hepatogenesis”, “FGF2 signaling during embryonic stem cell differentiation”, and “FGF signaling in pancreatic and hepatic differentiation of embryonic stem cells” (Fig. S1A–C), suggesting to be a potential indicator of cell differentiation that can be detected at serum level. Furthermore, albumin is a protein target of oxidation, protecting other proteins from oxidative damage. In our case, the high serum levels of albumin could also be associated with protection against cell oxidation, cell ageing and susceptibility to many other diseases. Experimental studies suggest that certain oxidation-sensitive proteins may act as antioxidant buffers to protect critical proteins against oxidation^27^.

Our proteomic analysis also highlighted the abundance of antithrombin III in serum after one year of nintedanib treatment^28^. This protein is one of the most important serine protease inhibitor in plasma where it regulates the clotting cascade by inhibiting thrombin, matriptase-3/TMPRSS7, and clotting factors IXa, Xa and XIa. Interestingly, researchers have found abnormalities in alveolar coagulation in acute and chronic lung injury, especially in systemic sclerosis and IPF, where a pro-clotting state with implications for clinical management^29^ has been demonstrated^30–33^. It is recognized that the physiological function of the clotting cascade extends beyond blood coagulation. Indeed, this cascade plays a pivotal role in inflammatory and repair responses to tissue injury. Uncontrolled coagulation activity contributes to the pathophysiology of various conditions, including inflammatory diseases and acute and chronic lung injury^34–36^. The up-regulation of serum ANT3, induced by nintedanib treatment, could have a protective role against a pro-coagulation environment. For this reason, nintedanib may interfere with regulation of the coagulation cascade, counteracting the progression of fibrosis.

The protein network also shows interaction between ANT3 and CREB1. It has been reported thrombin to induce expression of the c-fos gene (*c-fos*), activation of the (fos/jun) AP-1 site and expression of *Ccnd1* (cyclin D1), in precise correlation with the activation of CREB, leading to cell proliferation^37^. The abundance of antithrombin III found by us could counteract the concerted action of CREB1 and thrombin in cell proliferation. Our protein network also suggests that CREB 1 is part of a canonical pathway from PDGFR through PI3K and Akt to PPAR-γ. The same path from H-Ras could also activate MAPK signaling to PPAR-α. Both these paths lead to APOC3, which we found up-regulated in serum after one year of antifibrotic treatment. Both these pathways are known to be involved in the epithelial mesenchymal transition in cancer as well as fibrosis^38,39^. Salha *et al*. described these mechanisms as essential for mesenchymal cell transmigration through support of tumor growth, increase in power and contribution to tumor-angiogenesis^40^. From this point of view, up-regulation of APOC3 may be considered an expression of nintedanib activity and modulation of its targets in patients with IPF.

Although the interaction networks generated by this study are theoretical and future analyses by validation measures in a larger cohort of patients are needed to ascertain these interactions, this work has the advantage to shows, to the serum level, potential nintedanib molecular modulations.

## Conclusions

Nintedanib is a drug with anti-fibrotic, anti-proliferative and anti-inflammatory properties used in the treatment of lung cancer and IPF. Although some of its targets are well known (FGFR, VEGFR and PDGFR), other molecular mechanisms need to be further explored. Here, we used proteomics and bioinformatics to discover potential theranostic biomarkers at serum level in patients treated with nintedanib. Our differential proteomic approach identified 13 protein species that increased in abundance after one year of treatment. Enrichment analysis showed that all were related to the main targets of nintedanib (FGFR, VEGFR and PDGFR).

In particular, the abundance of HPT in serum could be potentially related to modulation of nintedanib targets that in turn interact with STAT3, STAT5 and CREB-binding protein. STAT3 is induced by VEGFR, FGFR and PDGFR and its mechanism of action is known to be related to cell differentiation in fibrosis. The abundance of albumin is related to cell differentiation processes by PDGFR, FGFR and c-Myc and may be an antioxidant buffer against oxidation in the lungs. Up-regulation of serum ANT3 could have a protective role against the pro-coagulation environment typical of IPF and could protect the lungs against thrombin-induced cell proliferation. Up-regulation of APOC3 is described as a possible result of inhibition of PPAR-γ and PPAR-α due to the effect of nintedanib on PDGFR. This inhibitory signal involves PI3K or MAPK, crucial molecules regulating epithelial mesenchymal transition, mesenchymal cell transmigration and cell proliferation in cancer as well as fibrosis.

The 13 differential protein species identified in serum of IPF patients after one year of treatment, and especially HPT, are candidate as potential theranostic markers of nintedanib action.

## Supplementary information

Supplementary information.

Supplementary information2.

Supplementary information3.

Supplementary information4.

Supplementary information5.

Supplementary information6.

Supplementary information7.

## References

[1] Cottin V, Capron F, Grenier P, Cordier J-F (2004). Diffuse idiopathic interstitial pneumonias. International multidisciplinary consensus classification by the American Thoracic Society and the European Respiratory Society, principal clinico-pathological entities, and diagnosis. Rev. Mal. Respir..

[2] Raghu G (2011). An official ATS/ERS/JRS/ALAT statement: idiopathic pulmonary fibrosis: evidence-based guidelines for diagnosis and management. Am. J. Respir. Crit. Care Med..

[3] Nathan SD (2017). Evaluating new treatment options. Am. J. Manag. Care.

[4] Hilberg F (2008). BIBF 1120: triple angiokinase inhibitor with sustained receptor blockade and good antitumor efficacy. Cancer Res..

[5] Hilberg F (2018). Triple Angiokinase Inhibitor Nintedanib Directly Inhibits Tumor Cell Growth and Induces Tumor Shrinkage via Blocking Oncogenic Receptor Tyrosine Kinases. J. Pharmacol. Exp. Ther..

[6] d’Alessandro M (2020). BAL biomarkers’ panel for differential diagnosis of interstitial lung diseases. Clin. Exp. Med..

[7] Brusasco V, Crapo R, Viegi G (2005). American Thoracic Society & European Respiratory Society. Coming together: the ATS/ERS consensus on clinical pulmonary function testing. Eur. Respir. J..

[8] Bianchi L (2013). A methodological and functional proteomic approach of human follicular fluid en route for oocyte quality evaluation. J. Proteom..

[9] Lehmann M (2018). Differential effects of Nintedanib and Pirfenidone on lung alveolar epithelial cell function in *ex vivo* murine and human lung tissue cultures of pulmonary fibrosis. Respir. Res..

[10] Yoon H-Y, Park S, Kim DS, Song JW (2018). Efficacy and safety of nintedanib in advanced idiopathic pulmonary fibrosis. Respir. Res..

[11] Grosso F, Roveta A, Gallizzi G, Belletti M (2018). Management of recurrent pleural mesothelioma: Successful rechallenge with nintedanib in combination with chemotherapy. Clin. Case Rep..

[12] Hong S-H (2019). Impact of Epidermal Growth Factor Receptor Mutation on Clinical Outcomes of Nintedanib Plus Docetaxel in Patients with Previously Treated Non-Small Cell Lung Cancer from the Korean Named Patient Program. Oncology.

[13] Raju SM (2019). Haptoglobin improves acute phase response and endotoxin tolerance in response to bacterial LPS. Immunol. Lett..

[14] Uskoković A (2012). STAT3/NF-κB interactions determine the level of haptoglobin expression in male rats exposed to dietary restriction and/or acute phase stimuli. Mol. Biol. Rep..

[15] Waters DW (2018). Fibroblast senescence in the pathology of idiopathic pulmonary fibrosis. Am. J. Physiol. Lung Cell Mol. Physiol..

[16] Waters DW (2019). STAT3 Regulates the Onset of Oxidant-induced Senescence in Lung Fibroblasts. *Am*. J. Respir. Cell Mol. Biol..

[17] Becerra A (2017). Endothelial fibrosis induced by suppressed STAT3 expression mediated by signaling involving the TGF-β1/ALK5/Smad pathway. Lab. Invest..

[18] Yu W (2017). β-Catenin Cooperates with CREB Binding Protein to Promote the Growth of Tumor Cells. Cell. Physiol. Biochem..

[19] Baarsma HA, Königshoff M (2017). ‘WNT-er is coming’: WNT signalling in chronic lung diseases. Thorax.

[20] Oda K (2016). Profibrotic role of WNT10A via TGF-β signaling in idiopathic pulmonary fibrosis. Respir. Res..

[21] Krishnamurthy N, Kurzrock R (2018). Targeting the Wnt/beta-catenin pathway in cancer: Update on effectors and inhibitors. Cancer Treat. Rev..

[22] Lanzarone, N. *et al*. Bronchoalveolar lavage and serum KL-6 concentrations in chronic hypersensitivity pneumonitis: correlations with radiological and immunological features. *Intern Emerg Med*., 10.1007/s11739-020-02281-8 (2020). 10.1007/s11739-020-02281-8 32078140

[23] Bergantini L (2019). Serial KL-6 analysis in patients with idiopathic pulmonary fibrosis treated with nintedanib. Respir. Investig..

[24] Chiba H, Otsuka M, Takahashi H (2018). Significance of molecular biomarkers in idiopathic pulmonary fibrosis: A mini review. Respir. Investig..

[25] Ball SG, Shuttleworth A, Kielty CM (2012). Inhibition of platelet-derived growth factor receptor signaling regulates Oct4 and Nanog expression, cell shape, and mesenchymal stem cell potency. Stem Cell.

[26] Erdélyi-Belle B (2015). Expression of Tight Junction Components in Hepatocyte-Like Cells Differentiated from Human Embryonic Stem Cells. Pathol. Oncol. Res..

[27] Rottoli P (2005). Carbonylated proteins in bronchoalveolar lavage of patients with sarcoidosis, pulmonary fibrosis associated with systemic sclerosis and idiopathic pulmonary fibrosis. Proteomics.

[28] Bergantini, L. *et al*. Antithrombin III as predictive indicator of survival in IPF patients treated with Nintedanib: a preliminary study. *Intern. Med. J*., 10.1111/imj.14768 (2020). 10.1111/imj.14768 32040256

[29] Tuinman PR, Dixon B, Levi M, Juffermans NP, Schultz MJ (2012). Nebulized anticoagulants for acute lung injury - a systematic review of preclinical and clinical investigations. Crit. Care.

[30] Collard HR (2010). Plasma biomarker profiles in acute exacerbation of idiopathic pulmonary fibrosis. Am. J. Physiol. Lung Cell Mol. Physiol..

[31] Scotton CJ (2009). Increased local expression of coagulation factor X contributes to the fibrotic response in human and murine lung injury. J. Clin. Invest..

[32] Markart P (2010). Safety and tolerability of inhaled heparin in idiopathic pulmonary fibrosis. J. Aerosol Med. Pulm. Drug. Deliv..

[33] Bargagli E (2014). Serum analysis of coagulation factors in IPF and NSIP. Inflammation.

[34] Landi C (2013). Towards a functional proteomics approach to the comprehension of idiopathic pulmonary fibrosis, sarcoidosis, systemic sclerosis and pulmonary Langerhans cell histiocytosis. J. Proteom..

[35] Landi C (2014). A system biology study of BALF from patients affected by idiopathic pulmonary fibrosis (IPF) and healthy controls. Proteom. Clin. Appl..

[36] Carleo A (2016). Comparative proteomic analysis of bronchoalveolar lavage of familial and sporadic cases of idiopathic pulmonary fibrosis. J. Breath. Res..

[37] Lee-Rivera I, López E, Parrales A, Alvarez-Arce A, López-Colomé AM (2015). Thrombin promotes the expression of Ccnd1 gene in RPE cells through the activation of converging signaling pathways. Exp. Eye Res..

[38] Liu Z (2019). Nuclear factor I/B promotes colorectal cancer cell proliferation, epithelial-mesenchymal transition and 5-fluorouracil resistance. Cancer Sci..

[39] Zhou X (2019). Cardamonin inhibits the proliferation and metastasis of non-small-cell lung cancer cells by suppressing the PI3K/Akt/mTOR pathway. Anticancer. Drugs.

[40] Salha S (2018). PDGF regulated migration of mesenchymal stem cells towards malignancy acts via the PI3K signaling pathway. Clin. Hemorheol. Microcirc..

