# Supplementary figures and images for: Idiopathic Pulmonary Fibrosis Serum proteomic analysis before and after nintedanib therapy
Source: Sci Rep. 2020 Jun 10;10:9378. doi: 10.1038/s41598-020-66296-z (PMC7287088; doi:10.1038/s41598-020-66296-z)

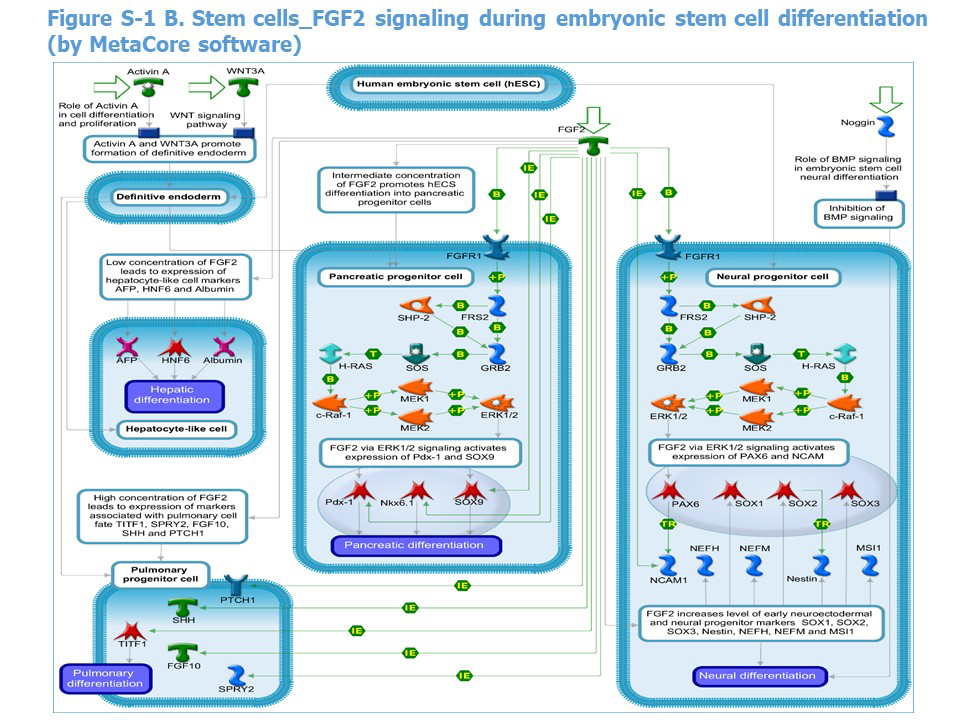

Supplement: Supplementary file 1 — Supplementary information. [file 41598_2020_66296_MOESM1_ESM.jpg]

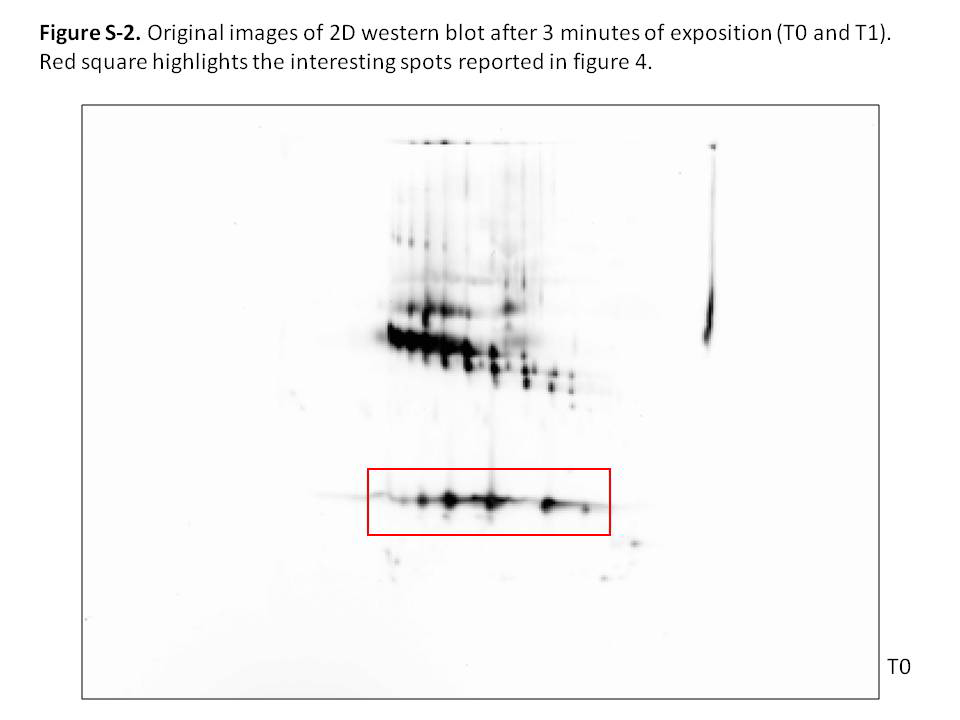

Supplement: Supplementary file 2 — Supplementary information2. [file 41598_2020_66296_MOESM2_ESM.jpg]

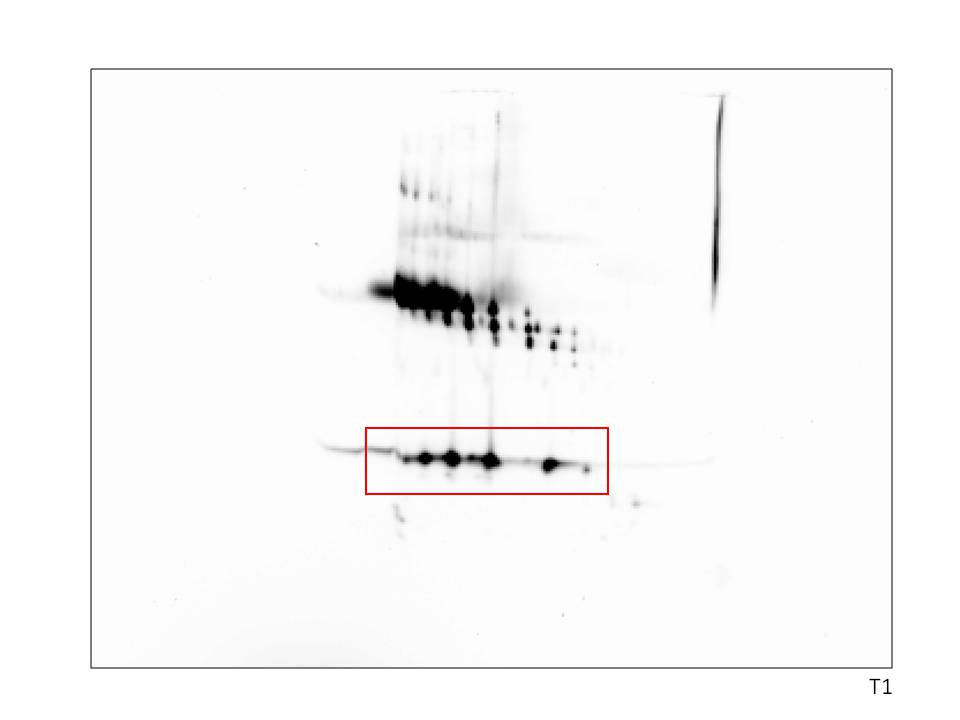

Supplement: Supplementary file 3 — Supplementary information3. [file 41598_2020_66296_MOESM3_ESM.jpg]

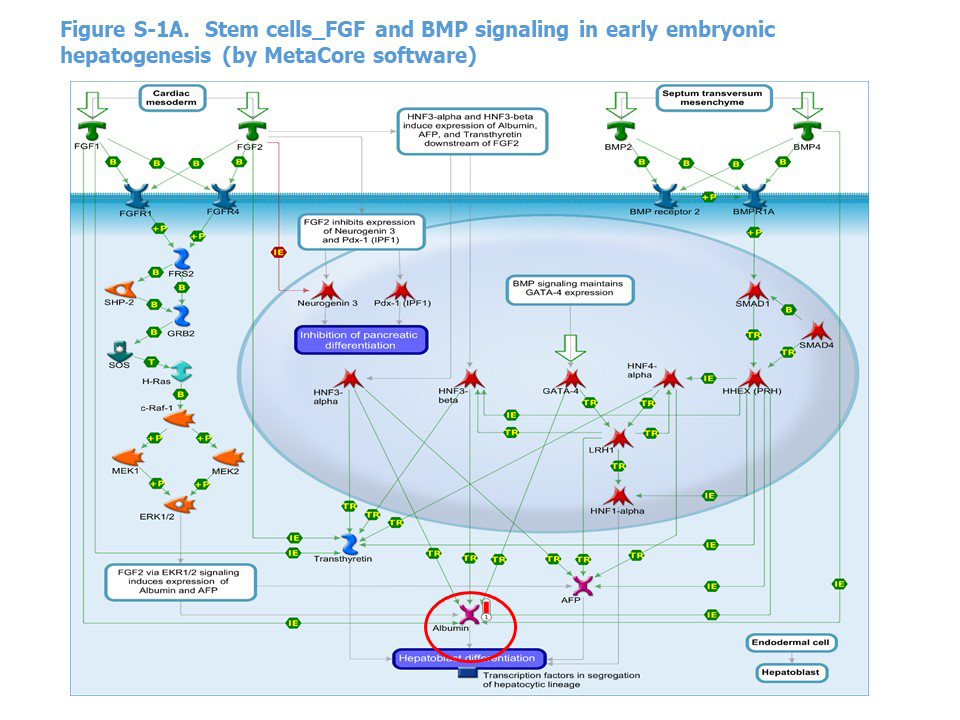

Supplement: Supplementary file 4 — Supplementary information4. [file 41598_2020_66296_MOESM4_ESM.jpg]

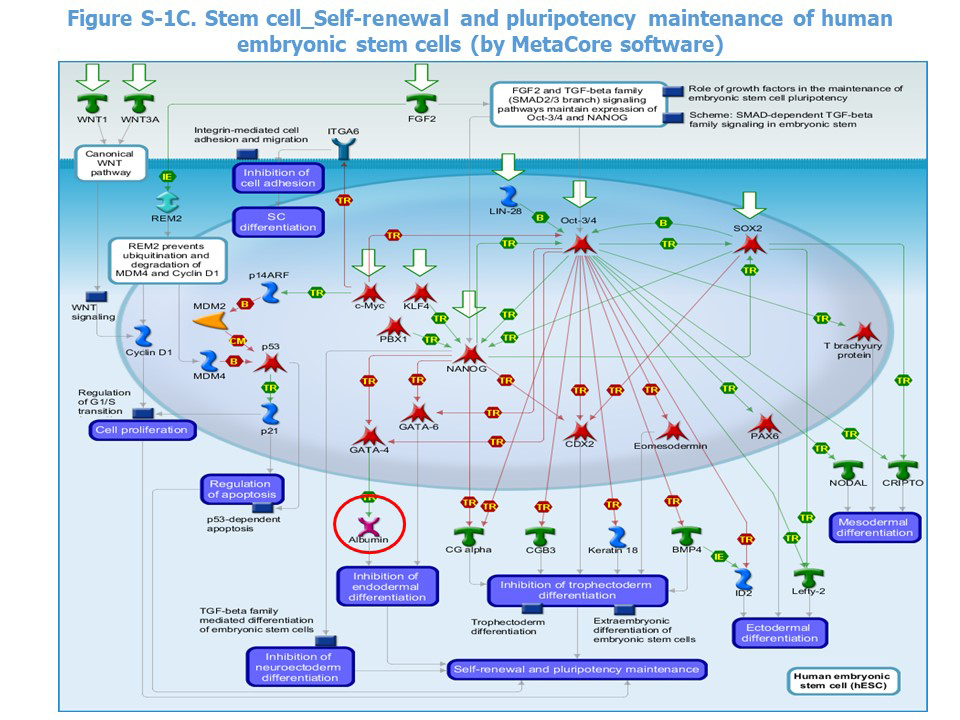

Supplement: Supplementary file 5 — Supplementary information5. [file 41598_2020_66296_MOESM5_ESM.jpg]

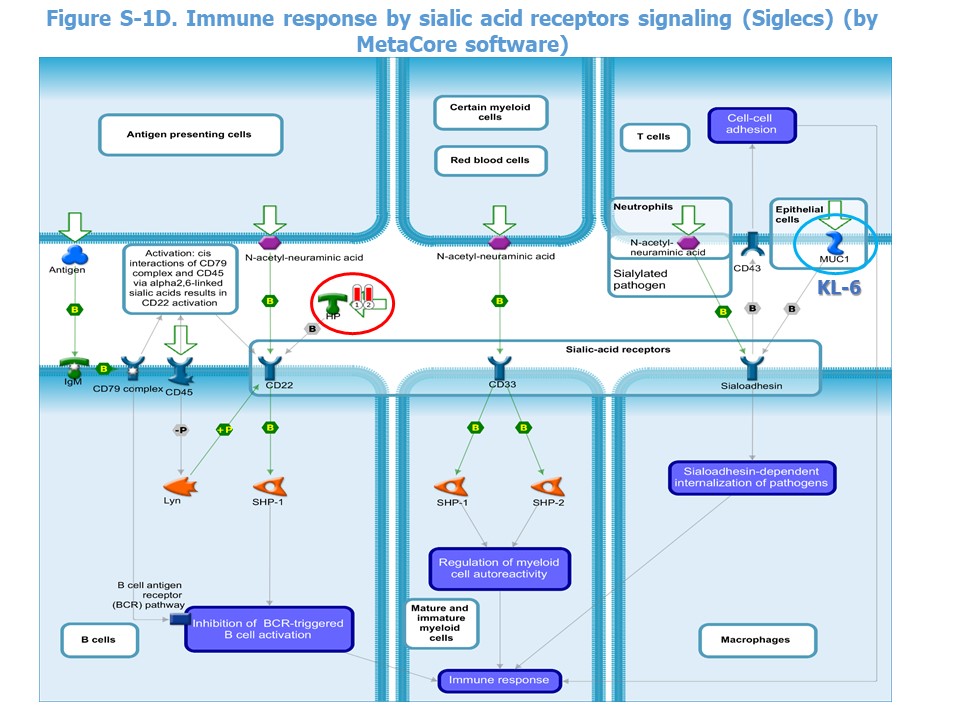

Supplement: Supplementary file 6 — Supplementary information6. [file 41598_2020_66296_MOESM6_ESM.jpg]
