# Supplementary material for: Idiopathic Pulmonary Fibrosis Serum proteomic analysis before and after nintedanib therapy
Source: Sci Rep. 2020 Jun 10;10:9378. doi: 10.1038/s41598-020-66296-z (PMC7287088; doi:10.1038/s41598-020-66296-z)
Supplement: Supplementary file 7 — Supplementary information7. [file 41598_2020_66296_MOESM7_ESM.docx]

**Idiopathic Pulmonary Fibrosis Serum proteomic analysis before and after nintedanib therapy**

Claudia Landi^1,2*^, Laura Bergantini^2^, Paolo Cameli^2^, Miriana d’Alessandro^2^, Alfonso Carleo^3^, Enxhi Shaba^1^, Paola Rottoli^2^, Luca Bini^1^, Elena Bargagli^2^.

^1^ Functional Proteomics Lab, Dept. Life Sciences, University of Siena, Italy

^2^ Respiratory Diseases and Lung Transplant Unit, Dept. Internal and Specialist Medicine, AOUS, Siena, Italy

^3^Department of Pneumology, Medical School Hannover (MHH), Hannover, Germany

**Supplemental Materials**

**Italian national drug inclusion criteria**

Patients with IPF were treated with the antifibrotic drug nintedanib according to Italian national drug inclusion/exclusion criteria. Nintedanib inclusion criteria in Italy are age ≥40 years, diagnosis of idiopathic pulmonary fibrosis according to international guidelines, FVC > 50% of predicted and DLCO > 30%. Exclusion criteria for nintedanib treatment in Italy are ALT, AST > 1.5 x ULN, total bilirubin > 1.5 x ULN, high risk of bleeding, INR > 2, PT, PTT > 150% of ULN, major surgery scheduled in the next 3 months or high risk of thrombosis.

**S-1 Serum preparation**

Blood was collected in the morning, after 8 hours of fasting, directly into serum tubes (BD vacutainer, SST II Advance, Plymouth UK) and centrifuged for 10 minutes at 1690 x*g*. The serum was recovered and stored at -80°C until analysis. For proteomic analysis, 10 μl of serum were diluted in 16 μl of buffer with 10% SDS w/v and 2,3% DTE w/v and successively heated until 95° for 7 min. Denaturing buffer containing 8M UREA w/v, 4% CHAPS w/v, 1% DTE w/v was added until to reach a final volume of 500 μl. Fifty microliters of this prepared sample contained 60 μg of proteins and were loaded by cup-loading on the IPGstrips for the analitical run. The remaining 450 μl of sample was used for the preparative run to the mass spectrometry analysis.

**S-2 Functional Proteomic analysis**

Two-dimensional electrophoresis was performed using the Immobiline–polyacrylamide system as described ^[1,2]^. Isoelectric Focusing (IEF) was carried out on non-linear wide range immobilized pH gradient (pH 3-10; 18 cm long IPG strips; GE Healthcare, Uppsala, Sweden) and using Ettan™ IPGphor™ system (GE Healthcare). IPG strips were rehydrated with 350 μl of the rehydration buffer (8M UREA w/v, 4% CHAPS w/v, 1% DTE w/v). Successively, the 100 μl of each sample, added with 0.2% (v/v) carrier ampholyte for analytical-runs and 2% (v/v) carrier ampholyte for MS-preparative runs were loaded by cup-loading with the cup applied at the cathodic end of the corresponding strip, on the Ettan IPGphor manifold (GE Healthcare). Isoelectric Focusing was performed under the following electrical conditions: 200 V for 8 h, a gradient until 3500V for 2h, a step of 3500V for other 2h. A gradient until 5000V for 2h and maintained 5000V for other 3h, another gradient until 8000V for 1h and a step of 8000V for 3h. In the end, is performed a gradient until 10000V for 1h and maintained for a total of 100,000 Vh. After the first dimensional run, the IPG gels were equilibrated in 6 M UREA, 2% w/v SDS, 2% w/v DTE, 30% v/v glycerol and 0.05 M Tris-HCl pH 6.8 for 12 min and for a further 5 min in the same solution where DTE was replaced by 2.5% w/v iodoacetamide and trace of bromophenol blue. The second dimension was carried out on 9-16% polyacrilamide linear gradient gels. Analytical gels were stained with ammonia silver nitrate, as previously described ^[3]^, while MS-preparative gels were stained using MS-compatible silver nitrate procedure as previously described ^[3,4]^. Silver stained gels were digitalized using an Image Scanner III laser densitometer supplied with the LabScan 6.0 software (GE Healthcare). Image analysis was carried out using Image Master 2D Platinum 7.0 software (GE Healthcare). For each tested condition, 6 different maps, from 6 patients, before and after 12 months of treatment with nintedanib, were analyzed. After spot detection and vertical and horizontal speckles and streaks removing, the analysis process was performed by matching all gels in each group with their reference gel having the best resolution and the greatest number of spots, chosen by the user and named “Master” by the software (intra-class analysis). Master reference gels were then matched with each other (inter-class analysis). According to a fold change of at least ±1.3 in percentage of relative volume (%V) (Vol: integration of optical density over the spot area; %V: Vol of a single spot divided by the total volume of spots computed over the whole image and expressed in percentage form), and to the statistical analysis by Wilcoxon test (p≤0.05) and FDR performed by RStudio Desktop 1.1.463 (Integrated Development for RStudio, Inc., Boston, USA, www.rstudio.com), differently abundant proteins have been found.

**Mass spectrometry by MALDI ToF-ToF**

Detected spot differences were successively identified by peptide mass fingerprinting on an Ultraflex III MALDI-TOF/TOF mass spectrometer (Bruker Daltonics, Billerica, MA). Protein spots from MS-compatible silver stained gels were manually excised, destained and dehydrated in acetonitrile. The spots were rehydrated by trypsin solution for protein digestion performed overnight at 37°C. 1.25 µl of each digested protein was spotted onto the MALDI target and allowed to dry. Then 0.75 μl of matrix solution (5mg/ml alpha-ciano 4 hydroxy cynnamic acid in 50% v/v Acetonitrile and 0.5% v/v trifluoroacetic acid) was applied to the dried sample and dried again. Trypsin digested peptides were MS analysed in the positive reflector mode according to defined parameters. The resulting mass lists were filtered for contaminant removal and mass fingerprinting searching was carried out using Mascot (Matrix Science Ltd., London, UK, http://www.matrixscience.com) on-line-available software. Mass fingerprinting search was carried out in Swiss-Prot/TrEMBL and NCBIprot databases, taxonomy was limited to Homo sapiens, mass tolerance was 100 ppm, and the acceptable number of missed cleavage sites was set at one. Alkylation of cysteine by carbamidomethylation was assumed and oxidation of methionine was considered as a possible modification.

**Multivariate analysis by Principal Component Analysis**

Principal Component Analysis (PCA) was performed by excel (analyze-it® module). In particular, the %V of each differentially abundant spot detected in each gel of the different conditions (T0 vs T1), were considered variables. These variables were organized in a specific matrix: the columns represented gel maps and the rows the differentially abundant spots. The matrix was loaded into the software producing a graphic representation of the results. PCA simplify the amount of data (%V variables) by linear transformation projecting the original variables into a new Cartesian system placing variables in a decreasing order. Variables with highest variance were projected on the first axis, the second on the second axis and so on. By this simplification it is possible to observe the distribution of each sample in a two-dimensional plane relies on the differential spots abundance in each CSF (spot maps).

**Cluster analysis**

Cluster analysis was performed by a free online software, Cluster 3.0. This analysis consented the visualization of the protein abundance trend in the two considered conditions (T0, T1). Spot numbers associated to the relative name of the identified proteins of the differentially abundant spots and their normalized mean of %V, were reported in a .txt file and uploaded on the software Cluster 3.0. In order to perform the analysis, on “adjust data” module has been selected “normalized gene” function. On “hierarchical” module was selected “cluster” and “Euclidean distance” functions. In the end, was selected “average linkage” as algorithm for cluster building. Cluster 3.0 software creates a file .cdt visualized thanks to “Java tree view” software. The final expression matrix reported the two conditions in the columns, the differential spots in the rows. The red and black colors represent the maximum and the minimum protein abundance, respectively.

**MetaCore analysis**

Identified proteins were processed by MetaCore 6.8 network building tool (GeneGo, St, Joneph, MI, USA) to perform protein network and pathways analysis. Gene names of the proteins were uploaded on MetaCore (http://portal.genego.com) that includes a manually annotated database of protein interactions and metabolic reactions obtained from the scientific literature. Thanks to the shortest-path algorithm it was possible to connect “nodes” representing proteins, through “arches”, representing protein interactions. The shortest-path algorithm links two uploaded experimental proteins through at maximum, a single node. Graphical representation reports proteins distributed in their subcellular environment. Enrichment analysis of the biological process was based on the hypergeometric distribution algorithm and relevant pathway maps, process networks and diseases (by biomarkers) were then prioritized according to their statistical significance (p<0,05).

**Western Blot analysis**

In order to validate the trustworthiness of 2DE results, bi-dimensional western blot for haptoglobin was performed. Aliquots of samples were prepared as previously described. Bi-dimensional WB was performed resolving 120 μg of proteins by 2DE for one patient per condition (T0, T1). Western blot preparative gels were electroblotted onto nitrocellulose by overnight transfer at a total current of 2 Å, at 4°C. Bi-dimensional membranes were reversibly stained with Ponceau Red (0.2% w/v Ponceau S in 3% w/v trichloroacetic acid) to confirm correct protein transfer. Hybridization with primary rabbit polyclonal anti-haptoglobin (Dakopatts, Glostrup Denmark) was carried out overnight at room temperature. Goat-anti-rabbit HRP-conjugate secondary antibodies (Sigma Aldrich), was incubated for 2 hours at room temperature. Immunostained spots were visualized by chemoluminescence by using ECL reagents (GE Healthcare) and Chemidoc instrument (Bio-Rad). Image acquisition was performed after 3, 6 and 9 minutes of exposition. After evaluations, we used the images at 3 minutes of exposition. Spot density was quantified by Image Master 2D Platinum.

**ELISA**

To analyze the secreted human haptoglobin level, serum samples from healthy subjects and IPF patients before and after 1 year of nintedanib treatment, were collected and we assessed using haptoglobin Quantikine ELISA Kit (R&D systems, Inc., Minneapolis, USA) according to the manufacturer’s instructions. The results were expressed in ng/ml.

**References**

1. Bjellqvist, B., Pasquali, C., Ravier, F., Sanchez, J.C., Hochstrasser, D. A nonlinear wide-range immobilized pH gradient for two-dimensional electrophoresis and its definition in a relevant pH scale. *Electrophoresis*.**14,**1357–65 (1993).

2. Hochstrasser, D.F., Harrington, M.G., Hochstrasser, A.C., Miller, M.J., Merril, C.R. Methods for increasing the resolution of two-dimensional protein electrophoresis. *Anal Biochem Sep*. **173**, 424–35 (1988).

3. Oakley, B.R., Kirsch, D.R., Morris, N.R. A simplified ultrasensitive silver stain for detecting proteins in polyacrylamide gels. *Anal Biochem*.**105,** 361–363 (1980).

4. Hochstrasser, D.F., Patchornik, A., Merril, C.R. Development of polyacrylamide gels that improve the separation of proteins and their detection by silver staining. *Anal Biochem*. **173**, 412–23 (1988).

5. Minter, M., Towbin, J., Harter, J., et al., Enzyme product blot for nondestructive assay of protein catalytic function in polyacrylamide gels. *Anal Biochem*. **178**(1), 22-26 (1989).
